# Study protocol: *Our Cultures Count*, the Mayi Kuwayu Study, a national longitudinal study of Aboriginal and Torres Strait Islander wellbeing

**DOI:** 10.1136/bmjopen-2018-023861

**Published:** 2018-06-27

**Authors:** Roxanne Jones, Katherine A Thurber, Jan Chapman, Catherine D’Este, Terry Dunbar, Mark Wenitong, Sandra J Eades, Lisa Strelein, Maureen Davey, Wei Du, Anna Olsen, Janet K Smylie, Emily Banks, Raymond Lovett

**Affiliations:** 1 National Centre for Epidemiology and Population Health, Research School of Population Health, The Australian National University, Acton, Australia; 2 University of Adelaide, Adelaide, South Australia, Australia; 3 Apunipima Cape York Health Council, Bungalow, Queensland, Australia; 4 Baker Heart and Diabetes Institute, Melbourne, Victoria, Australia; 5 Australian Institute of Aboriginal and Torres Strait Islander Studies, Acton, Australia; 6 Tasmanian Aboriginal Centre, Hobart, Tasmania, Australia; 7 Centre for Research on Inner City Health, Saint Michael’s Hospital, Toronto, Ontario, Canada; 8 Sax Institute, Haymarket, New South Wales, Australia

**Keywords:** culture, aboriginal and torres strait islander, indigenous population, longitudinal studies, social determinants of health

## Abstract

**Introduction:**

Aboriginal and Torres Strait Islander peoples are Australia’s first peoples and have been connected to the land for ≥65 000 years. Their enduring cultures and values are considered critical to health and wellbeing, alongside physical, psychological and social factors. We currently lack large-scale data that adequately represent the experiences of Aboriginal and Torres Strait Islander people; the absence of evidence on cultural practice and expression is particularly striking, given its foundational importance to wellbeing.

**Method and analysis:**

Mayi Kuwayu: The National Study of Aboriginal and Torres Strait Islander Wellbeing (Mayi Kuwayu Study) will be a large-scale, national longitudinal study of Aboriginal and Torres Strait Islander adults, with linkage to health-related administrative records. The baseline survey was developed through extensive community consultation, and includes items on: cultural practice and expression, sociodemographic factors, health and wellbeing, health behaviours, experiences and environments, and family support and connection. The baseline survey will be mailed to 200 000 Aboriginal and Torres Strait Islander adults (≥16 years), yielding an estimated 16 000–40 000 participants, supplemented through face-to-face recruitment. Follow-up surveys will be conducted every 3–5 years, or as funding allows. The Mayi Kuwayu Study will contribute to filling key evidence gaps, including quantifying the contribution of cultural factors to wellbeing, alongside standard elements of health and risk.

**Ethics and dissemination:**

This study has received approval from national Human Research Ethics Committees, and from State and Territory committees, including relevant Aboriginal and Torres Strait Islander organisations. The study was developed and is conducted in partnership with Aboriginal and Torres Strait Islander organisations across states and territories. It will provide an enduring and shared infrastructure to underpin programme and policy development, based on measures and values important to Aboriginal and Torres Strait Islander peoples. Approved researchers can access confidentialised data and disseminate findings according to study data access and governance protocols.

**Strengths and limitations of this study**

- Mayi Kuwayu: The National Study of Aboriginal and Torres Strait Islander Wellbeing (the Mayi Kuwayu Study) will be a large-scale, national longitudinal study of Indigenous Australian adults, with linkage to health-related administrative records.
- With an estimated minimum 16 000 participants, the study will be an order of magnitude larger than any previous prospective study of Aboriginal and Torres Strait Islander adults.
- The baseline survey was developed through consultations with individuals and communities across Australia, and includes items on: cultural practice and expression, sociodemographic factors, health and wellbeing, health behaviours, experiences and environments, and family support and connection.
- The Mayi Kuwayu Study will be an exemplar of Aboriginal and Torres Strait Islander research governance.
- The Mayi Kuwayu Study is not intended to be population representative; the aim of the study is to generate evidence based on internal comparisons, and to examine within-population variation.

## Introduction

### Rationale

Aboriginal and Torres Strait Islander peoples are Australia’s first peoples and have been connected to the land for at least the last 65 000 years.^1^ Aboriginal and Torres Strait Islander cultures and values remain strong in contemporary Australia and are celebrated as among the longest continuing cultures in the world.

Broadly, culture may comprise the ideas and self-concepts of a group (eg, artefacts, attitudes, beliefs, customs, norms, symbols and values) and the lived practice and expression of these in differing contexts. Culture also includes historical events and standards of behaviour that evolve and change over time.^2^ According to the literature, key Aboriginal and Torres Strait Islander cultural domains may include: knowledges and beliefs, cultural expressions, country and caring for country (referring to the essential relationship between a people and their traditional territories), language, self-determination and family, kinship and community.^3^

From a holistic perspective, culture can be considered a foundational component of, and contributor to, health and wellbeing, in addition to physical, psychological and social factors. Despite the potential importance of culture to health and wellbeing, there is a paucity of research exploring the association between culture and wellbeing among Aboriginal and Torres Strait Islander peoples.^4^ Further, the potential mechanisms through which culture impacts on wellbeing (and vice versa) remain unknown. Aboriginal and Torres Strait Islander culture is increasingly being recognised as a critical, yet under-researched, determinant of health by communities, organisations and policy-makers.^5 6^ There is a clear need for research that identifies how Aboriginal and Torres Strait Islander peoples navigate and express the differing cultures in which their lives exist, and how this relates to their health and wellbeing.

Mayi Kuwayu: The National Study of Aboriginal and Torres Strait Islander Wellbeing (Mayi Kuwayu Study) is designed to address this lack of knowledge on a national scale, generating evidence regarding culture and its relationship to health and wellbeing. ‘Mayi Kuwayu’ means ‘to follow Aboriginal people over a long time’ in Ngiyampaa language (language of the Wongaibon peoples of New South Wales, Australia). This data resource will help us to understand the role of culture in health and wellbeing. In addition, the study will be an order of magnitude larger than any previous prospective study of Aboriginal and Torres Strait Islander adults, enabling the generation of robust, needed evidence on health and wellbeing.

This study arose from the need to quantify what has been written about and often described as instinctive to many Aboriginal and Torres Strait Islander people: cultures and their relationship to wellbeing.^7^ As such, this study is designed to privilege Aboriginal and Torres Strait Islander views and experiences. This study employs measures of culture that have been codeveloped with a diversity of Aboriginal and Torres Strait Islander peoples through an iterative qualitative process. The Mayi Kuwayu Study employs a salutogenic framework, enabling identification of cultural and other assets that promote wellbeing.^8^

The Mayi Kuwayu Study aims to examine health and wellbeing in relation to cultural practice and expression, taking into account the varied contexts in which Aboriginal and Torres Strait Islander peoples live. This study was developed primarily within a social epidemiology framework, concerned with the social structures, institutions and relationships that influence health and wellbeing. Culture may impact wellbeing directly, indirectly through social determinants of health and/or through other pathways.^9^

### Objectives

The primary aim of this study is to enable quantification of associations and pathways between cultural practice and expression, social determinants of health, health behaviours, and health and wellbeing outcomes for Aboriginal and Torres Strait Islander peoples. Specifically, the project will generate: (1) indicators of Aboriginal and Torres Strait Islander cultural practice and expression that capture diversity and maintain meaning across contexts; (2) large-scale data on cultural practice and expression, sociodemographic factors, health and wellbeing, health behaviours, experiences and environments, and family support and connection, both cross-sectionally and over time and, (3) a state-of-the-art data resource for investigating Aboriginal and Torres Strait Islander wellbeing, which can also serve as a framework for policy and programme planning.

The primary study hypothesis is that cultural practice and expression (eg, connection to country, language use, kinship ties) is associated with health and wellbeing. Understanding and quantifying these associations could inform policy, for example, by supporting programme development that appropriately accounts for and promotes cultural engagement, in order to promote wellbeing. This evidence could also inform individual behaviour; for example, it might encourage cultural engagement and revitalisation by individuals and communities, which could in turn lead to an improvement in wellbeing.

## Methods

### Study recruitment

**Figure 1:**
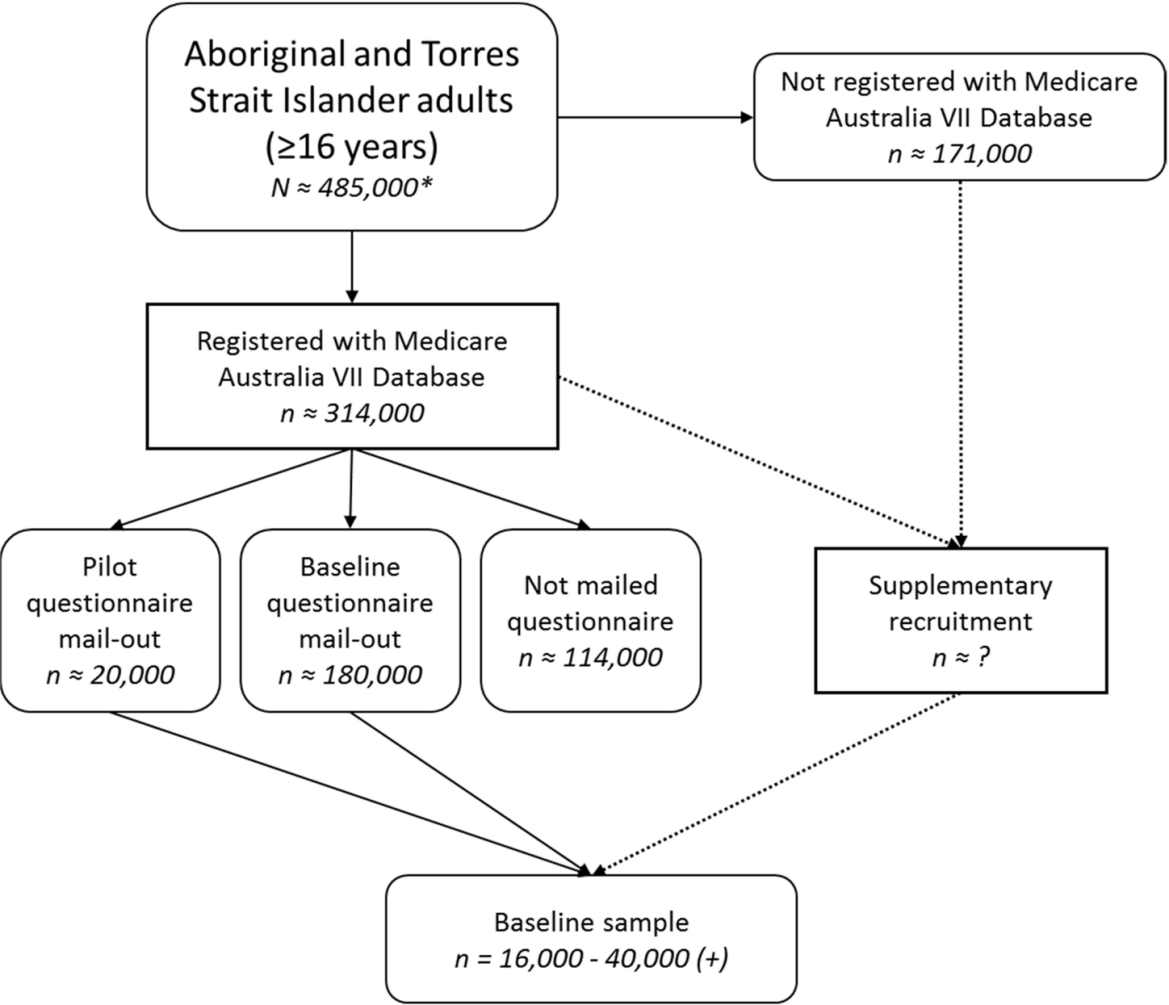
Flow diagram for Mayi Kuwayu Study recruitment. *Estimated undercount-adjusted population of Aboriginal and Torres Strait Islander peoples aged ≥16 years. Calculated by adjusting the raw Census count (n=414 532) for the overall 2016 Census undercount for the Aboriginal and Torres Strait Islander population (17.5%).^10^ Data are unavailable on the extent of undercount by age group within the Aboriginal and Torres Strait Islander population. VII, Voluntary Indigenous Indicator.

All Aboriginal and Torres Strait Islander people aged 16 years and older living in Australia are eligible to participate (figure 1).

#### Primary sampling frame

The intended primary recruitment method for this study is through a mail-out to people registered as Aboriginal and/or Torres Strait Islander in the Medicare Australia database. Medicare Australia is the national healthcare administration database, including all Australian citizens and permanent residents. Aboriginal and Torres Strait Islander people who choose to self-identify as Indigenous in the Medicare Australia database are recorded through the Medicare Australia Voluntary Indigenous Indicator (VII); this ‘Medicare VII database’ constitutes the primary sampling frame for this study.

We estimate that the Medicare VII database represents 68% of all Aboriginal and Torres Strait Islander people in Australia (total population=786 689, based on undercount-adjusted Census data^10^). As at October 2017, the Medicare VII database included 533 832 self-identified Aboriginal and Torres Strait Islander peoples, 313 732 of whom are in the eligible age range. The age and sex distribution of Aboriginal and Torres Strait Islander peoples self-identified in the Medicare VII database closely mirrors that of the total Aboriginal and Torres Strait Islander population (table 1).

**Table 1:** Age and sex distribution of Aboriginal and Torres Strait Islander peoples in the Medicare VII database (VII) and in the overall population

Age group	Males				Females				Persons			
	VII		Census		VII		Census		VII		Census	
	# (1000’s)	%	# (1000’s)	%	# (1000’s)	%	# (1000’s)	%	# (1000’s)	%	# (1000’s)	%
16–24	38.1	11.2	56.0	13.5	41.8	12.3	53.5	12.9	79.9	23.5	109.5	26.4
25–34	39.6	11.2	44.0	10.6	43.1	12.3	44.8	10.8	82.7	23.5	88.9	21.4
35–49	35.3	11.2	51.6	12.4	38.8	12.3	56.8	13.7	74.1	23.5	108.4	26.2
≥50	36.5	11.6	50.2	12.1	41.6	13.2	57.6	13.9	78.1	24.8	107.8	26.0
Total (≥16 years)	149.4	47.5	201.8	48.7	165.4	52.5	212.7	51.3	314.7	100.0	414.5	100.0

Medicare VII database (‘VII’) data presented in this table includes persons registered on the Medicare VII database and aged 16 and over; persons missing age are excluded from total (n=314 732).

Census data presented in this table reflect raw 2016 Census population counts for Aboriginal and Torres Strait Islander people aged 16 years and over (n=414 532).^30^ Data are not adjusted for undercount, as data are unavailable on the extent of undercount by age group and sex within the Aboriginal and Torres Strait Islander population.

VII, Voluntary Indigenous Indicator.

The Department of Human Services extract a mailing list from the Medicare VII database, and use this mailing list to distribute survey materials (information sheet, consent form and baseline survey) to potential participants. Previous Australian studies have sampled from the total Australian population (using the full Medicare Australia database, not restricted to those self-identified as Indigenous through the VII)^11 12^ and have recruited substantial numbers of Aboriginal and Torres Strait Islander people through this process (n=1985).^11^ However, no studies to date have purposely sampled the Aboriginal and Torres Strait Islander population using the Medicare VII database.

We intend for Mayi Kuwayu Study materials to be distributed to a mailing list of 200 000 people from the Medicare VII database. Previous mail-out surveys using the Medicare Australia database in the total Australian population have achieved response rates of 18%–44%.^11 12^ Given potential additional barriers to recruiting Aboriginal and Torres Strait Islander participants (such as increased mobility, respondent burden, mistrust in research), we conservatively estimate a response rate of 8%–20%, which would result in a total of 16 000–40 000 participants through the primary recruitment method.

The aim will be to achieve a baseline sample that aligns with the population distribution across age group (16–24; 25–34; 35–49; ≥50 years), sex (male; female) and remoteness (major cities; inner and outer regional areas; remote and very remote). To achieve this, we will use stratified sampling, with strata based on the sex, age group and remoteness categories specified above. We will conduct a pilot mail-out of 20 000 surveys to estimate response rate for each stratum (age group by sex by remoteness). This will inform the extent to which strata need to be oversampled in the main mail-out, allowing for differential response rates by age, sex and remoteness, to achieve the desired sample distribution, and a minimum of 500 participants in each stratum.

The study materials distributed in the pilot mail-out will match the materials distributed in the full mail-out. Surveys completed in the pilot study phase will be included in the baseline data collection.

All participants who receive a survey through the pilot and main mail-out will have the option to complete the paper-based survey or to complete the survey online or over the phone.

#### Supplementary recruitment methods

While the Medicare VII database will serve as the primary sampling frame for the baseline survey, participation in the Mayi Kuwayu Study is not restricted to those who are registered with the Medicare VII database. All Aboriginal and/or Torres Strait Islander persons aged 16 years or over are eligible to participate, regardless of whether or not they receive a survey through the mail-out process.

Field testing during the development phase indicated that face-to-face (versus self-complete) delivery supported participation by those with low literacy levels. Therefore, supplementary recruitment will occur through face-to-face surveying in selected areas expressing an interest, and/or areas demonstrating low response rates or high levels of missing data on completed surveys in the pilot mail-out. All participants recruited through the supplementary recruitment method will also have the option to complete the survey online or over the phone.

Supplementary recruitment may also occur through study promotion (such as advertising via social media and through local community-controlled organisations and word of mouth). Any eligible person can complete the survey online or over the phone, or contact the Mayi Kuwayu Study to request a paper survey.

Given that this recruitment method potentially enables participants to complete the survey multiple times, baseline data will be checked for duplicates based on name, address and other identifying information.

### Study components

**Figure 2:**
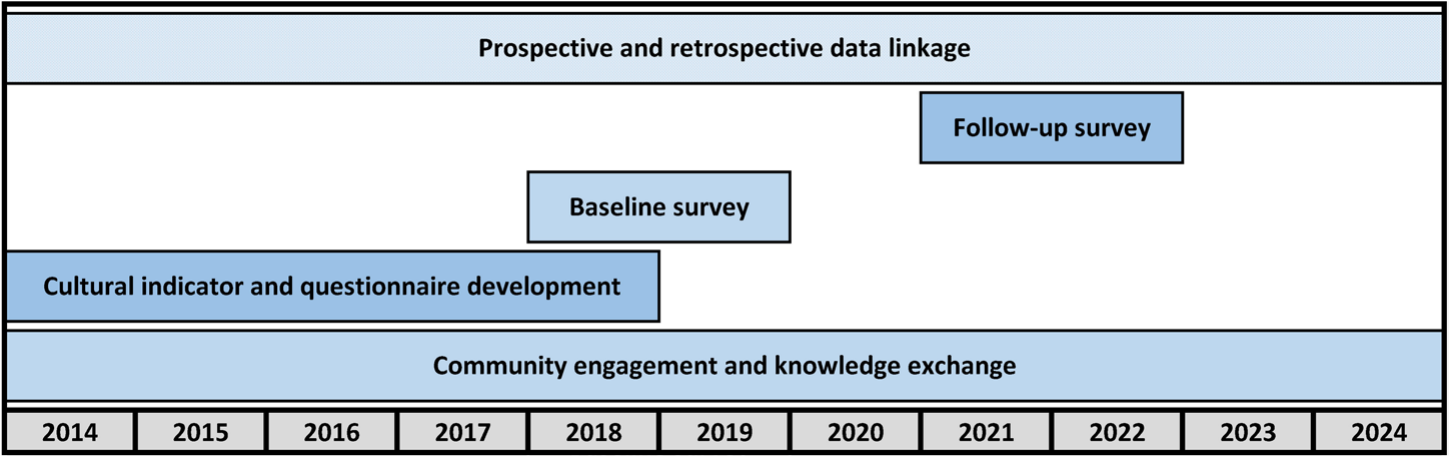
Mayi Kuwayu Study timeline.

The Mayi Kuwayu Study encompasses four main components: (1) cultural indicator and survey development; (2) baseline survey (including pilot mail-out); (3) repeat follow-up surveys and (4) data linkage, which are briefly outlined below(figure 2).

#### Cultural indicator and survey development

Survey items, including indicators of Aboriginal and Torres Strait Islander culture, have been developed through reviewing the literature and through consultation with a total of 165 Aboriginal and Torres Strait Islander peoples attending 24 focus groups across Australia from 2014 to 2017. Participants in this process were aged 16 to >70 years and represent a diversity of contexts and lived experiences. Early versions of the Mayi Kuwayu baseline survey were pilot tested with 160 and 209 Aboriginal and Torres Strait Islander participants in two ‘proof-of-concept’ studies, respectively.

The iterative processes of developing and refining the cultural indicators and other survey items will be detailed elsewhere. This process was critical to developing appropriate and meaningful questions about culture, to enable quantification of cultural expressions and contexts, and their associations with health and wellbeing outcomes, across diverse settings.

#### Baseline survey

The baseline survey will contain survey items on cultural practice and expressions, sociodemographic factors, health and wellbeing, health behaviours, experiences and environments, and family support and connection. Key survey themes within each domain are summarised in table 2. All survey items included in the survey are based on established instruments, modified instruments or instruments developed through the community consultation process.

**Table 2:** Key domains and measures included in the baseline survey

Domain	Theme
Cultural practice and expression	Country and connection to country, Indigenous beliefs and knowledge, cultural expression, self-determination and leadership, language, family, kinship and community, identity
Sociodemographic factors	Age, sex, housing, education, employment, financial situation, household composition
Health and wellbeing	Life satisfaction, health status, health conditions, medication use, social and emotional wellbeing, functional limitation
Health behaviours	Physical activity, alcohol and tobacco use, health service use
Experiences and environments	Services in the community, experiences of racism, community safety, environmental conditions, life events
Family support and connection	Family cohesion and connectedness, caring for others, stolen generation

To enable data linkage and recontact for follow-up surveys, the baseline survey will collect data on participants’ first and last name, postal address, phone number and email address. In addition, the baseline survey will collect contact details for an additional family member or friend (close contact) who can be approached to provide information to assist in recontacting the participant if required.

#### Follow-up surveys

Participants in the baseline survey will be followed up by survey every 3–5 years, or as funding allows. Follow-up surveys will maintain the core components of the baseline survey, with potential for addition or removal of survey items depending on priority and resourcing. Follow-up surveys will be distributed directly to participants (mail, email or phone). Participants will be able to nominate their preferred method of contact on the baseline survey.

#### Data linkage

Retrospective and continuing prospective linkage of baseline survey data to health-related records will provide ongoing outcome data independent of resurvey. The types of linkage datasets pursued will include hospitalisation, cancer registrations, deaths and disease notifications.

### Patient and public involvement statement

The Mayi Kuwayu Study arose from community-identified priorities, and has been developed through extensive consultation; these processes have enabled the generation of a survey that can meaningfully and appropriately collect data about Aboriginal and Torres Strait Islander cultures and wellbeing across diverse settings.

Study governance mechanisms will ensure that engagement is ongoing throughout the implementation of the baseline and follow-up surveys, and during research question prioritisation, data analysis and interpretation. In addition to supporting the generation of meaningful results, this will support ongoing cohort retention. For example, participants can nominate to receive study newsletters, and appropriate social and Aboriginal and Torres Strait Islander media and forums will be used to inform participants of study progress and key outcomes.

The Mayi Kuwayu Study will create a collaborative resource governed by Aboriginal and Torres Strait Islander organisations, researchers and communities. The study is Indigenous led, with direct involvement from Aboriginal and Torres Strait Islander researchers (including the Study’s lead) who are leaders in their field and who are well respected in their communities, bringing a depth of experience and accountability to ensure adherence to appropriate community protocols. The governance structure will ensure that Aboriginal and Torres Strait Islander peoples and partner organisations from across Australia are involved at every level and every stage of the project.

## Statistical methods

Statistical analyses will involve both cross-sectional and longitudinal methods. This will include estimates of prevalence, incidence and changes over time of cultural and health risk factors and health and wellbeing outcomes, and examination of their inter-relationships at baseline and over time. We will examine whether cultural or other factors moderate or mediate associations between risk factors and health outcomes. Analyses will be informed by a conceptual framework developed for each research question and include a range of methods appropriate for cross-sectional, longitudinal, and linked data and causal modelling, adjusting for correlation of measures within individual over time and missing data, where appropriate.

We conservatively estimate that at least 16 000–40 000 Indigenous adults will participate in the baseline survey, based on the expected 8%–20% response rate to baseline survey mail-out and supplementary recruitment. All participants in the baseline survey will be included for data linkage. If there is 20% loss to follow-up, there will be 12 800–32 000 participants with longitudinal survey data.

For analyses of the prevalence, incidence and changes over time for cultural, health or other factors, the study will allow highly precise estimation within sex and 5-year age groups, with 95% CI for the smallest group within ±1.0%–3.5% and 0.1 SDs for proportions and means, respectively. For cross-sectional and longitudinal analyses, the study will have at least 80% power, with a 5% significance level, to detect sex-specific ORs of 1.2–1.3 for binary outcomes and HRs of 1.2–1.4 for time-to-event analyses, for exposures of ≥10% prevalence and binary outcomes of 2%–5% prevalence.

## Ethics and dissemination

Development, recruitment, retention and dissemination strategies used in the Mayi Kuwayu Study are based on principles of Indigenous data sovereignty and best practice for cohort studies of Aboriginal and Torres Strait Islander peoples.^13–16^ It is intended that the study be perceived as an Indigenous community activity that promotes Indigeneity, building on Indigenous relationality and the importance of family and kin networks.

### Consent

Participation in the Mayi Kuwayu Study is voluntary. Potential participants will receive a plain-language information sheet about the study, along with the consent form and survey. The study uses a structured, staged consent form, where participants opt-in to specific study components (baseline survey, data linkage and/or recontact), rather than a blanket consent form covering all study components. This empowers individuals to participate only in aspects of the study with which they are comfortable. This staged approach has been previously used in an Aboriginal and Torres Strait Islander cohort study, and has been demonstrated to be an appropriate method for recruiting and retaining Aboriginal study participants.^17^ Participants can withdraw consent at any time.

### Dissemination

Dissemination of findings from the Mayi Kuwayu Study will be subject to approval from the governing body. With approval, findings will be disseminated through forms including community dissemination meetings, community reports and feedback sheets, policy briefs, manuscripts for peer-reviewed publication, conference presentations and public seminars.

## Discussion and implications

### Representativeness

The Mayi Kuwayu Study is not intended to be representative of the entire Aboriginal and Torres Strait Islander adult population. However, the study aims to capture much of the diversity of the Aboriginal and Torres Strait Islander population nationally, with sufficient heterogeneity across exposures. The aim of the study is to generate evidence based on internal comparisons, and to examine within-population variation in these associations.

While this has not been explored specifically within the Aboriginal and Torres Strait Islander population, in general, representativeness is not necessary for reliable quantification of exposure–outcome relationships.^18 19^ Algebraic work and simulation studies provide evidence on the validity of internal comparisons in the face of varying response rates.^20^ Moreover, experience over time from a wide range of epidemiological research has also shown this to be the case. For example, the British Doctors’ Study, where doctors are clearly not representative of the general population, yet findings based on internal comparisons remain valid (that is, the association between smoking and mortality)^21^; pooled analyses incorporating cohort studies, case–control and other study designs tend to find materially similar findings among studies with varying response rates.^22^ Further, a representative sample may not contain sufficient numbers of specific exposures or outcomes of importance. Thus, while high response rates and representativeness are essential to censuses and population health surveys (where the main aim is to accurately estimate point prevalence), these features are not essential or recommended for cohort studies.^23^

### Implications

The Mayi Kuwayu Study will establish an ethical, community-focused and Aboriginal-controlled resource that will contribute to a holistic and robust understanding of Aboriginal and Torres Strait Islander culture, health and wellbeing. It will be an Aboriginal-controlled collaborative resource for research, conducted in strict accordance with current ethical, community and Aboriginal and Torres Strait Islander research standards.^24–29^ The participatory methods will support the relevance of findings for individuals, communities, health services and policy-makers across portfolios.

This novel study will be the first of its kind, providing a large-scale national cohort study about the wellbeing of Aboriginal and Torres Strait Islander adults. It will provide the first community-derived measures of culture, and the first quantitative evidence regarding Indigenous cultural expressions and contexts at the national level. It will enable the first large-scale investigation of the relationship between culture and wellbeing for Aboriginal and Torres Strait Islander adults. This will identify opportunities to incorporate culture in programmes and policy to improve Aboriginal and Torres Strait Islander wellbeing.

There is currently limited incorporation of Aboriginal and Torres Strait Islander culture in programmes and policies—a dimension that is likely to be critical to effectiveness and acceptability. Evidence from the Mayi Kuwayu Study may increase the prioritisation of culture in the design of programme and policy.

## Supplementary Material

Reviewer comments

Author's manuscript

## References

[1] Clarkson C, Jacobs Z, Marwick B, et al Human occupation of northern Australia by 65,000 years ago. Nature 2017;547:306–10. 10.1038/nature22968 28726833

[2] Bessant J, Watts R Sociology Australia. 3rd ed Crows Nest, NSW: A&U Academic, 2007.

[3] Salmon M, Gilbert R, Dance P, et al Defining the indefinable: Descriptors of Indigenous Peoples’ Cultures and their links to Health and Wellbeing: ANU Press In Press.

[4] Bourke S, Wright A, Guthrie J, et al Evidence review of Indigenous culture for health and wellbeing. Journal of Health, Wellness and Society. In Press.

[5] Department of Health and Ageing. National Aboriginal and Torres Strait Islander Health Plan (NATSIHP) 2013-2023. Canberra: Australian Government, 2013.

[6] Department of Health. Implementation Plan for the National Aboriginal and Torres Strait Islander Health Plan 2013-2023. Canberra: Australian Government, 2015.

[7] Oscar J Culture, Relationships, Health: Human Rights in Practice. Indigenous Allied Health Australia National Conference Perth, 2017.

[8] Antonovsky A The salutogenic model as a theory to guide health promotion. Health Promot Int 1996;11:11–18. 10.1093/heapro/11.1.11

[9] Berkman L, Kawachi I, Glymour M Social Epidemiology. Second Edition New York: Oxford University Press, 2014.

[10] Australian Bureau of Statistics. 2940.0 - Census of Population and Housing: Details of Overcount and Undercount, Australia. Canberra: ACT 2017, 2016 http://www.abs.gov.au/AUSSTATS/abs@.nsf/Lookup/2940.0 (accessed 1 Feb 2018).

[11] Banks E, Redman S, Jorm L, et al Cohort profile: the 45 and up study. Int J Epidemiol 2008;37:941–7. 10.1093/ije/dym184 17881411 PMC2557061

[12] Lee C, Dobson AJ, Brown WJ, et al Cohort Profile: the Australian Longitudinal Study on Women’s Health. Int J Epidemiol 2005;34:987–91. 10.1093/ije/dyi098 15894591

[13] Study of Environment of Child Health Study Investigators. The Study of Environment on Aboriginal Resilience and Child Health (SEARCH): study protocol. BMC Public Health 2010;10:287. 20507632 10.1186/1471-2458-10-287 PMC2896939

[14] Thurber KA, Banks E, Banwell C; LSIC Team. Cohort profile: footprints in time, the Australian longitudinal study of Indigenous Children. Int J Epidemiol 2015;44:dyu122 10.1093/ije/dyu122 PMC4521121 25011454

[15] Sayers SM, Mackerras D, Singh G, et al An Australian Aboriginal birth cohort: a unique resource for a life course study of an Indigenous population. A study protocol. BMC Int Health Hum Rights 2003;3:1 10.1186/1472-698X-3-1 12659639 PMC152651

[16] Thurber KA, Olsen A, Guthrie J, et al; ’Telling our story. creating our own history’: caregivers’ reasons for participating in an Australian longitudinal Study of Indigenous children. Under Review. 10.1186/s12939-018-0858-1 PMC6138915 30219069

[17] Sayers S, Singh G, Mackerras D, et al Australian Aboriginal Birth Cohort study: follow-up processes at 20 years. BMC Int Health Hum Rights 2009;9:23 10.1186/1472-698X-9-23 19775475 PMC2761846

[18] Mealing NM, Banks E, Jorm LR, et al Investigation of relative risk estimates from studies of the same population with contrasting response rates and designs. BMC Med Res Methodol 2010;10:26 10.1186/1471-2288-10-26 20356408 PMC2868856

[19] Boffetta P Internal and external validity of cohort studies. Ann Agric Environ Med 2011;18:283–4. 22216799

[20] Ponsonby A-L, Dwyer T, Couper D Is this finding relevant? Generalisation and epidemiology. Aust N Z J Public Health 1996;20:54–6. 10.1111/j.1467-842X.1996.tb01336.x 8799067

[21] Doll R, Peto R, Boreham J, et al Mortality in relation to smoking: 50 years' observations on male British doctors. BMJ 2004;328:1519–28. 10.1136/bmj.38142.554479.AE 15213107 PMC437139

[22] Collaborative Group on Hormonal Factors in Breast Cancer. Breast cancer and hormonal contraceptives: collaborative reanalysis of individual data on 53 297 women with breast cancer and 100 239 women without breast cancer from 54 epidemiological studies. Lancet 1996;347:1713–27. 8656904 10.1016/s0140-6736(96)90806-5

[23] Rothman KJ, Gallacher JE, Hatch EE Why representativeness should be avoided. Int J Epidemiol 2013;42:1012–4. 10.1093/ije/dys223 24062287 PMC3888189

[24] Willows N Ethical principles of health research involving Indigenous peoples. Appl Physiol Nutr Metab 2013;38:iii–v. 10.1139/apnm-2013-0381 24053533

[25] Smylie J The ethics of research involving Canada’s Aboriginal populations. CMAJ 2005;172:977–77. 10.1503/cmaj.1041676 15824389 PMC556019

[26] National Health and Medical Research Council. Values and ethics: guidelines for ethical conduct in Aboriginal and Torres Strait Islander research. Canberra, 2003.

[27] Australian Institute of Aboriginal and Torres Strait Islander Studies Guidelines for Ethical Research in Australian Indigenous Studies. 2nd ed Canberra, 2011.

[28] Dunbar T, Scrimgeour M Ethics in Indigenous Research – Connecting with Community. J Bioeth Inq 2006;3:179–85. 10.1007/s11673-006-9018-1

[29] Anderson I Ethics and health research in Aboriginal communities. Sydney: Allen and Unwin, 1996:153–64.

[30] Australian Bureau of Statistics. 2071.0 - Census of Population and Housing: Reflecting Australia - Stories from the Census, 2016 - Aboriginal and Torres Strait Islander Population. Canberra: ACT 2017, 2017 www.abs.gov.au/ausstats/abs@.nsf/mf/2071.0 (accessed 1 Feb 2018).

