# Supplementary material for: Study protocol: Our Cultures Count, the Mayi Kuwayu Study, a national longitudinal study of Aboriginal and Torres Strait Islander wellbeing
Source: BMJ Open. 2018 Jun 27;8(6):e023861. doi: 10.1136/bmjopen-2018-023861 (PMC6020975; doi:10.1136/bmjopen-2018-023861)
Supplement: Reviewer comments [file bmjopen-2018-023861.reviewer_comments.pdf]

PEER REVIEW HISTORY

BMJ Open publishes all reviews undertaken for accepted manuscripts. Reviewers are asked to complete a checklist review form (**http://bmjopen.bmj.com/site/about/resources/checklist.pdf**) and are provided with free text boxes to elaborate on their assessment. These free text comments are reproduced below.

ARTICLE DETAILS

**TITLE (PROVISIONAL)**	Study Protocol: Our Cultures Count -- The Mayi Kuwayu Study, a national longitudinal study of Aboriginal and Torres Strait Islander wellbeing
**AUTHORS**	Jones, Roxanne; Thurber, Katherine; Chapman, Jan; D'Este, Catherine; Dunbar, Terry; Wenitong, Mark; Eades, Sandra; Strelein, Lisa; Davey, Maureen; Du, Wei; Olsen, A; Smylie, Janet; Banks, Emily; Lovett, Raymond

This article was not externally reviewed at BMJ Open. Protocols that have been independently assessed prior to submission to BMJ Open are usually fast-tracked to publication on the grounds that further substantial changes will not be possible. This independent assessment will usually be external, independent review for both a substantial grant award from a non-commercial or government funder and ethics approval.
